# Symmetry breaking and effects of nutrient walkway in time-dependent bone remodeling incorporating poroelasticity

**DOI:** 10.1007/s10237-022-01573-6

**Published:** 2022-04-08

**Authors:** L. Esposito, V. Minutolo, P. Gargiulo, M. Fraldi

**Affiliations:** 1 Department Engineering, University of Campania “Luigi Vanvitelli”, Aversa, Italy; 2 Institute for Biomedical and Neural Engineering, Reykjavík University, Reykjavík, Iceland; 3 Department of Science, Landspítali Hospital, Reykjavík, Iceland; 4 Department of Structures for Engineering and Architecture, University of Napoli “Federico II”, Napoli, Italy

**Keywords:** Bone tissue, Bone remodeling, Poroelasticity, Porous media, Fluid flow

## Abstract

Bone is an extraordinary biological material that continuously adapts its hierarchical microstructure to respond to static and dynamic loads for offering optimal mechanical features, in terms of stiffness and toughness, across different scales, from the sub-microscopic constituents within osteons—where the cyclic activity of osteoblasts, osteoclasts, and osteocytes redesigns shape and percentage of mineral crystals and collagen fibers—up to the macroscopic level, with growth and remodeling processes that modify the architecture of both compact and porous bone districts. Despite the intrinsic complexity of the bone mechanobiology, involving coupling phenomena of micro-damage, nutrients supply driven by fluid flowing throughout hierarchical networks, and cells turnover, successful models and numerical algorithms have been presented in the literature to predict, at the macroscale, how bone remodels under mechanical stimuli, a fundamental issue in many medical applications such as optimization of femur prostheses and diagnosis of the risk fracture. Within this framework, one of the most classical strategies employed in the studies is the so-called Stanford’s law, which allows uploading the effect of the time-dependent load-induced stress stimulus into a biomechanical model to guess the bone structure evolution. In the present work, we generalize this approach by introducing the bone poroelasticity, thus incorporating in the model the role of the fluid content that, by driving nutrients and contributing to the removal of wastes of bone tissue cells, synergistically interacts with the classical stress fields to change homeostasis states, local saturation conditions, and reorients the bone density rate, in this way affecting growth and remodeling. Through two paradigmatic example applications, i.e. a cylindrical slice with internal prescribed displacements idealizing a tract of femoral diaphysis pushed out by the pressure exerted by a femur prosthesis and a bone element in a form of a bent beam, it is highlighted that the present model is capable to catch more realistically both the transition between spongy and cortical regions and the expected non-symmetrical evolution of bone tissue density in the medium–long term, unpredictable with the standard approach. A real study case of a femur is also considered at the end in order to show the effectiveness of the proposed remodeling algorithm.

## Introduction

The bone tissue is a dynamic system able to modify dynamically its outer shape and inner microstructure in response to chemo-mechanical stimuli coming from the environment, through several processes such as growth (mass change), remodeling (mass redistribution associated to changes in material properties), and morphogenesis (shape and structural modifications).

From a biological point of view, only at the end of the last century (Frost 1969; Martin 1984; Turner 1991, 1998), a description of the activities of the different bone cell populations was formalized, clarifying some key mechanisms like that of formation and resorption of the bone tissue due to osteoblasts and osteoclasts cells. In particular, remodeling of bone tissue occurs at the trabecular surface by the collaborative cellular activities of bone-resorbing osteoclasts and bone-forming osteoblasts (Parfitt 1994). Osteocytes, the most abundant cell type in bone tissue, are postulated to orchestrate the metabolic activities of these effector cells (Tatsumi et al. 2007; Nakashima et al. 2011). Although the exact mechanism by which osteocytes perceive mechanical load remains unclear, osteocytes are believed to be the most likely mechanosensory cells (Cowin et al. 1991, 2007; Mullender and Huiskes 1997; Huo et al. 2008; Adachi et al. 2009a,b), forming a complex intercellular network—the lacuno-canalicular system—via slender cell processes housed in the porosities inside the mineralized bone matrix (Kamioka et al. 2001, 2009; Sugawara et al. 2005). This intercellular network system acts as the pathway of mechanical signals from the osteocytes to the cells on the trabecular surfaces (Adachi et al., 2009c).

From a biomechanical point of view, the bone remodeling phenomenon involves changes in trabecular architecture, apparent density, and, in turn, modifications of the material properties. The first studies were based on clinical observations. Galileo Galilei compared the dimensions of bones from animals of different sizes and suggested that their forms were determined by their functions, gravity, and environment (Ascenzi 1993; Minutolo et al., 2020). Wilhelm Roux in 1880 was the first author to introduce the concept of functional adaptation of the bone tissue to describe growth and remodeling in response to a changing environment (Fung 1990). Wolff (1869, 1892, 1986) developed the trajectorial hypothesis—known as the *Wolff law*—i.e., the alignment of the trabeculae of the cancellous bone to the principal stress directions.

To date, many theories and mathematical models have been proposed by several authors to describe the remodeling phenomenon (Ambrosi et al., 2019; Oumghar et al. 2020; Della Corte et al., 2020). As said, it is known, at least since the end of the nineteenth century, that the loading history affects the growth and remodeling processes of the bone (Frost 1987), a relevant problem for the mathematical modeling being to capture, in a realistic and computable way, the interaction between mechanical loading and bone evolution. This has been generally attempted by means of a stimulus function linking mechanical and physiologic parameters involved in the bone tissue processes.

Two general approaches are proposed in the literature: the *adaptive elasticity theory* and the *bone maintenance theory*. The adaptive elasticity theory, developed for the first time by Cowin and Hegedus (1976), is a continuum mechanical-based formulation, in which the remodeling equations relate the change of the bone tissue density to mechanical stimuli (Huiskes 1987; Jang and Kim 2008). In particular, Cowin et al. (1992) proposed a mathematically rigorous theory for the remodeling of the internal architecture of the cancellous bone, called the *evolutionary Wolff's law*. The tissue bone was supposed to be a poroelastic biphasic material consisting of a solid phase, comprising bone cells and extracellular matrix, and a fluid phase, identified as the extracellular fluid. Moreover, Cowin (1986) introduced the Fabric Tensor, a second-order tensor to describe the anisotropy of the bone tissue, which was also employed as an evolving variable responsible for the tissue remodeling and leading to a state of equilibrium as stress and Fabric eigenvectors coincide.

The bone maintenance theory was developed by Fyhrie and Carter (1986) and accounted for both adaptations of bone apparent density and trabecular architecture. This theory is based on optimizing a local remodeling objective function depending on the apparent density, the orientation of material axes, and the stress tensor. The optimization analysis showed that trabecular orientations aligned with the principal stress trajectories. Expanding these ideas, Carter et al. (1987) proposed a method to account for the entire stress history of a bone, by introducing the daily remodeling stimulus. A key finding of this study was that the solution did not converge to a unique equilibrium state. On the basis of this behavior, Carter et al. (1989) later suggested the existence of an attractor state, rather than a remodeling equilibrium state. Starting from this, Beaupré et al. (1990a) extended bone maintenance theory including time-dependent remodeling and adding surface growth, with internal remodeling considered as surface growth in internal cavities. The model proposed by Beaupré et al. (1990a)—the so-called *Stanford law*—will be deeply discussed in the next section.

Apart from the use of the theory of elasticity to describe remodeling—by using Wolff law, modified similar relations as well as introducing multiscale models (Matsuura et al., 2003; Li et al., 2007; Coelho et al., 2009; Perrin et al., 2019)—the influence of interstitial fluid on bone remodeling plays a crucial role, often highlighted in literature with respect to processes occurring at cellular level (Fornells et al., 2007; You et al., 2008). The complex network of lacunae-canaliculi channels in the bone tissue (Li et al., 1987; Grimm and Williams 1997; Nauman et al., 1999; Lim and Hong 2000; Beno et al. 2006) permits interstitial fluid flow through micro-porosities (Piekarski and Munro 1977; Weinbaum et al., 1994; Cowin et al., 1995). Being the porosity of the inter-trabecular matrix greater than that of the lacunar-canalicular system (Cowin 1999), the interstitial fluid from the lacunar-canalicular channels can flow into and out of these larger inter-trabecular porosities, which acts as low-pressure reservoirs. The literature reports that fluid flow enhances cell proliferation (Jiang et al. 2002; Kapur et al., 2003) and the expression of phenotypic markers of osteoblastic cells (Owan et al., 1997; You et al., 2001; Wu et al., 2006). Also, it seems to promote the release of the paracrine factors necessary for the anabolic response of bone to mechanical loads (Forwood 1996; Baker et al., 2001; Gnetos et al., 2005; Li et al., 2005). Moreover, fluid flow was as well shown to increase osteocytic prostaglandins (Ajubi et al., 1996, 1999) and nitric oxide (Klein-Nulend et al., 1995) and is believed to play important roles in providing nutrients and removing wastes, being directly involved in cellular mechano-transduction (Burger and Klein-Nulend 1999; Bonewald and Johnson 2008; Fritton and Weinbaum 2009). Several authors have suggested that when a whole bone is deformed, the strain-induced pressure gradient will cause bone fluid to flow in the pericellular matrix space of the lacunar-canalicular system, inducing a drag force on the matrix fibers (Weinbaum et al., 1994; Knothe Tate 2003; Cowin 2007). As a consequence, the coupling between fluid flow and different stress and strain measures was deeply investigated (Kameo et al., 2016a; Kuman Tiwari 2017; Crevacuore et al., 2019), at different scales (Ganesh et al. 2020) and by including cyclic loadings (Kameo et al., 2016b; Li et al., 2020).

At the cellular level, many authors suggest that the osteocyte is the cell best situated to perceive physical signals, especially interstitial fluid flow (Weinbaum et al., 1994; Duncan and Turner 1995; Turner et al. 1994). Sanchez et al. (2020) and Giorgio et al. (2019) focused their works on the role of fluid flow in bone mechanobiology. Sandino et al. (2017), by modeling in a poroelastic numerical framework 76 cubes of trabecular bones, quantified the variation in the mechanical stimuli bone due to the presence of the fluid flow. Hence, the flow is considered as a stimulus that drives osteocyte response, since pressure variation within the bone fluid can lead to shear stress forces, hydrostatic pressure and electric field at the cell membrane of the osteocytes. These mechanisms stimulate osteocytes to produce and secrete hormonal and biochemical messengers such as cytokines and growth factors which affect cell differentiation and proliferation, therefore regulating the bone remodeling process (Pollack et al., 1977; Kelly et al. 1985; Montgomery et al. 1988; Reich et al., 1990; Rubin et al. 1997; Jacobs et al., 1998; Jin et al. 2021).

By starting from this framework, the present study is aimed to extend the Stanford law proposed by Beaupré et al. (1990b) by including the dimensionless fluid content, being the bone tissue supposed to be more realistically as poroelastic. As it will be shown, this parameter affects the relation between stress stimulus at the tissue level and the rate of density responsible for the remodeling, regulating the nutrients supply and thus the activity of bone tissue cells. In other words, in our model, the stress stimulus at tissue level was supposed to be a function of the density, the stress, and the fluid content as well. As a consequence, if the fluid content increases, the rate of the bone formation increases, and vice versa, this cooperating with the stress to break some symmetries in the outcome of the bone remodeling. Finally, the remodeling rate relation was also assumed to provide both a zone in which the bone is in a homeostatic state and a zone of saturation. In particular, the zone of homeostatic activity, the so-called Dead Zone (DZ), as proposed by Beaupre et al. (1990b), corresponds to a range of mechanical stimulus (around a reference value that bone senses as a physiological stimulus) within which no net change of bone density is provided, the saturation zone being instead the region where the rate of density remains constant for an increment or decrement of the stress stimulus, in the case of bone formation or absorption, respectively. This assumption is related to the evidence reported by Adams et al. (1997) in the bone remodeling response to high mechanical stimuli.

In what follows, it is first recalled the Stanford law, the governing equations of poroelasticity, the proposal of isotropic model influenced by the fluid flow and some selected examples of remodeling (Sect. 2). Then, results obtained by applying the proposed model are compared with standard ones, to highlight how poroelasticity contributes to determine non-symmetrical bone density profiles even if in presence of symmetry of both geometry and loads, as actually can be observed in vivo (Sect. 3). Finally, Sect. 4 is devoted to concluding remarks and perspectives.

## Materials and methods

### Isotropic remodeling model—the Stanford law

Beauprè et al. (1990a) proposed a unified time-dependent approach for periosteal and internal bone remodeling that takes into account the amount of bone surface area on which osteoclasts and osteoblasts can operate. They extended the bone maintenance theory developed by Carter and colleagues (Carter 1987; Fyhrie and Carter 1986; Whalen et al., 1988) into a time-dependent remodeling theory. The essence of this approach is that the bone tissue needs a certain level of mechanical stimulus to maintain itself. If the bone tissue experiences exceeding stimulation, additional bone will be deposited. On the other hand, if bone tissue is insufficiently stimulated, it will be resorbed. The proper level is set by systemic and local biochemical influences of adjacent tissues. The difference between this appropriate level and the actual imposed one of daily mechanical stimulation determines the impetus and speed of remodeling. The model proposed by Beauprè et al. (1990a)—known as the Stanford law—is isotropic, and the bone remodeling response is expressed as a function of the mechanical stimulus, the so-called daily stress stimulus.

At the macroscopic scale, the bone adaptation process is described on a daily basis by relating the remodeling rate to a set of stresses corresponding to successive loading conditions. The daily stress stimulus measured at the continuum level, $\psi$, is defined as:

1 $$\psi = \left( {\sum\limits_{day} {n_{i} \overline{\sigma }_{i}^{m} } } \right)^{\frac{1}{m}}$$

where $n_{i}$ is the number of cycles of load $i$, $m$ is a constant and $\overline{\sigma }_{i} = \sqrt {2EU_{i} }$ is the local effective stress. Note that $E$ represents Young’s modulus and $U_{i}$ is the Strain Energy Density produced by the load $i$ in a certain point. The constant $m$ is a weighting factor for the relative importance of the stress magnitude and the number of load cycles. For $m = 1$, the stress magnitude and the number of cycles are equally important. Increasing values of $m$ indicate an increasing dependence on those activities having high-stress magnitudes.

At the microscopic level, the bone remodeling response is expressed by the daily stress stimulus at the tissue level, $\psi_{t}$, related to $\psi$ by:

2 $$\psi_{t} = \left( {\frac{{\hat{\rho }}}{\rho }} \right)^{2} \psi$$

being $\rho$ the apparent density and $\hat{\rho } = 2.283\,{g \mathord{\left/ {\vphantom {g {cm^{3} }}} \right. \kern-\nulldelimiterspace} {cm^{3} }}$ the corresponding value of the bone at maximum density, assumed equal to the density of the fully mineralized tissue (Beauprè et al., 1990b).

The remodeling response is measured in terms of the bone resorption/formation rate, $\dot{r}\left( {{{\mu m} \mathord{\left/ {\vphantom {{\mu m} {day}}} \right. \kern-\nulldelimiterspace} {day}}} \right)\;$, which gives the net tissue volume formed or resorbed per unit time and reference area. The remodeling rate relation is typically nonlinear and spatially inhomogeneous (Carter 1982; Cowin and Hegedus 1976; Frost 1986; Huiskes et al., 1987), depending in fact on the bone tissue type and the sites. It is reasonable to assume the possible existence of a homeostatic region corresponding to a range of “normal” activities, and absorption and formation zones associated with decreased and increased levels of daily stress stimulus, respectively.

**Fig. 1:**
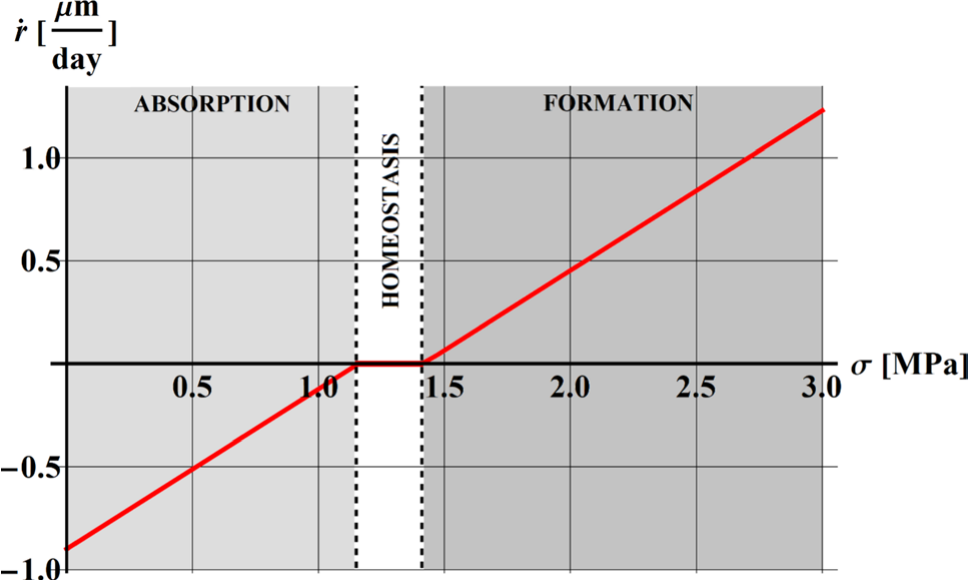
The remodeling rate $\dot{r}$ as a function of the stress, by keeping fixed the density to the value $\rho = 1\;{{\text{g}} \mathord{\left/ {\vphantom {{\text{g}} {{\text{cm}}^{3} }}} \right. \kern-\nulldelimiterspace} {{\text{cm}}^{3} }}$

In the study by Beauprè et al. (1990b), the remodeling rate, $\dot{r}$, is assumed to be linear and related to the difference between the daily stress stimulus at tissue level, $\psi_{t}$, and a reference value, $\psi_{ref}$, by the following piecewise function:

3 $$\dot{r} = \left\{ {\begin{array}{*{20}l} {c_{f} \left( {\psi_{t} - \psi_{ref} - w} \right)} \hfill & {if\,\psi_{t} > \psi_{ref} + w} \hfill & {{\text{formation}}} \hfill \\ 0 \hfill & {if\,\psi_{ref} + w < \psi_{t} < \psi_{ref} - w} \hfill & {{\text{homeostasis}}} \hfill \\ { - c_{r} \left( {\psi_{ref} - \psi_{t} - w} \right)} \hfill & {if\,\psi_{t} < \psi_{ref} - w} \hfill & {{\text{absorption}}} \hfill \\ \end{array} } \right.$$

where $c_{r}$ and $c_{f}$ are the slopes of the resorption and formation ramps, respectively. The slope of the bone resorption curve, $c_{r}$, is typically greater than the slope related to the bone apposition, $c_{f}$, being the osteoclastic activity faster than the osteoblastic one (Frost 1986; Parfitt 1983). Figure 1 shows the remodeling rate, $\dot{r}$, as a function of the stress, for a value of the apparent density equal to $\rho = 1\;{{\text{g}} \mathord{\left/ {\vphantom {{\text{g}} {{\text{cm}}^{3} }}} \right. \kern-\nulldelimiterspace} {{\text{cm}}^{3} }}$.

The equation (3) represents the relation that can be used to calculate the external remodeling and the rate of tissue apposition or resorption on external surfaces (periosteum and endosteum). Furthermore, the linear relation (3) exhibits the so-called Dead Zone (DZ), the homeostatic region that is a range of stimulus of width $2w$ around the reference stimulus, $\psi_{ref}$, within which no net bone mass change is produced.

On the basis of bone histomorphometry (Frost 1983), Beauprè et al. (1990a) calculated the internal remodeling by relating the density change rate, $\dot{\rho }$, with the bone surface area per unit volume, $S_{v}$, by:

4 $$\dot{\rho } = \dot{r}S_{v} \hat{\rho }$$

The specific surface area, $S_{v}$, was empirically determined as a function of the bone porosity by Martin (1984) for both cortical and cancellous bone, spanning the entire range of apparent density. The relation proposed by this study was a non-uniform function of the apparent density, with intermediate values of the specific area greater than those corresponding to extremely porous or dense bone, being the bone surface area differently available for osteoblastic and osteoclastic actions.

Summing up, it is generally accepted a time-dependent theory for bone remodeling in which internal and external remodeling are treated in a unified and consistent fashion as a surface-mediated phenomenon (Beauprè et al., 1990a). External remodeling changes (changes in shape or size) are then calculated directly from the linear rate of bone apposition or resorption on periosteal surfaces. Internal remodeling instead results from the linear bone apposition and resorption on internal surfaces. The local bone specific surface area provides the key link between the linear remodeling rate and the rate of change of bone apparent density.

### Poroelasticity constitutive equations

As it is well known, Terzaghi (1943) first proposes a model for the one-dimensional consolidation in order to analyze the influence of pore fluid flow on soil deformation. Biot (1935, 1941) was instead the first author who proposed a complete theory of linear poroelasticity, introducing the concept of the effective medium approach, which is able to describe the coupling between the deformation of linear elastic materials, governed by the classical Hooke’s law, and the interstitial fluid flow, ruled by the Darcy’s law (see also Esposito et al., 2022 for some non-standard behaviours). Two further rigorous approaches, i.e., the mixture theory and the homogenization derivations, have been presented in the literature, considering different averaging processes, all leading, however, to an equivalent set of governing equations.

By following the effective medium approach, the poroelastic field variables are assumed to be the stress measures, i.e., the total stress tensor ${{\varvec{\upsigma}}}$ and the pore pressure $p$, and the strain measures, i.e., the strain in the solid phase ${{\varvec{\upvarepsilon}}}$ and the dimensionless variation in fluid content $\zeta$. In particular, the variation in fluid content $\zeta$ is the variation of the fluid per unit volume of the porous material due to diffusive fluid mass transport.

The Darcy’s law is a form of the balance of linear momentum relating the fluid mass flow rate vector, ${\mathbf{q}}$, and to the gradient of the pore pressure $p$, according to the following equation

5 $${\mathbf{q}} = - \kappa \,\nabla p$$

where $\kappa$ is the coefficient of permeability, expressed as the ratio between the intrinsic permeability $k$ and the fluid viscosity $\mu$, the intrinsic permeability being only function of the porous structure.

It can be remarked that the porous media can be in drained or undrained conditions. Hence, the constitutive parameters of the material are governed by:

- the undrained moduli, indicated by the subscript *u*, corresponding to the situation in which the pores are filled by the fluid whose flow is constrained;
- the drained elastic moduli, indicated with no subscript, corresponding to the situation in which the pore fluid is fully drained and the fluid flow is allowed.

The stress–strain relationship for a poroelastic isotropic material can be written in the form:

6 $${{\varvec{\upsigma}}} + \alpha p{\mathbf{I}} = 2G{{\varvec{\upepsilon}}} + \frac{2G\nu }{{1 - 2\nu }}{\text{tr}} ({{\varvec{\upepsilon}}}){\mathbf{I}}$$

where $G$ and $\nu$ are the drained isotropic elastic constants, i.e., the shear modulus and the Poisson’s ratio, respectively, ${\text{tr}} ({{\varvec{\upepsilon}}})$ is the trace of the strain tensor and ${\mathbf{I}}$ is the identity tensor.

The relationship between the dimensionless fluid content $\zeta$ and the stress is:

7 $$2G\zeta = \alpha \frac{1 - 2\nu }{{1 + \nu }}\left( {{\text{tr}} ({{\varvec{\upsigma}}}) + \frac{3}{B}p} \right)$$

where $\alpha$ is the Biot-Willis parameter, and $B$ is the Skempton coefficient. Note that, if the pore pressure vanishes, Eqs. (2) and (7) give ${\text{tr}} ({{\varvec{\upsigma}}}) = 2G\frac{1 + \nu }{{1 - 2\nu }}{\text{tr}} ({{\varvec{\upepsilon}}})$ and $\zeta = \alpha \,{\text{tr}} ({{\varvec{\upepsilon}}})$, respectively. Thus, $\alpha$ is the ratio of the fluid volume gained (or lost) in a material element due to the volume change of that element when loaded under the drained condition.

Nur and Byerlee (1971) provided an expression for the coefficient $\alpha$, as:

8 $$\alpha = 1 - \frac{K}{{K_{s} }}$$

being $K$ and $K_{s}$ the drained bulk modulus and the one of the solid phase, respectively.

By setting the undrained condition ($\zeta = 0$) in Eq. (7), the pressure results:

9 $$p = - \frac{B}{3}{\text{tr}} ({{\varvec{\upsigma}}})$$

Thus, the Skempton coefficient $B$ measures how the stress is partitioned between solid and fluid phases; moreover, taking into account Eq. (4), the parameter $B$ results to be related to the drained and undrained solid properties and the fluid properties by means of the expression:

10 $$B = \frac{{\alpha K_{f} }}{{\left[ {\alpha - \phi \left( {1 - \alpha } \right)} \right]K_{f} + \phi K}} = \frac{{3\left( {\nu_{u} - \nu } \right)}}{{\alpha \left( {1 - 2\nu } \right)\left( {1 + \nu_{u} } \right)}}$$

being $K_{f}$ and $\phi$ the bulk modulus of the fluid and the porosity, respectively.

The value of *B* tends to 1 for completely saturated materials (i.e., the hydrostatic stress is carried by the fluid that completely fills the pores); on the contrary, for *B* = 0 the pore pressure vanishes and the stress is completely carried by the solid skeleton.

The undrained elastic bulk and Poisson moduli can be expressed as:

11 $$K_{u} = K\left[ {1 + \frac{{\alpha^{2} K_{f} }}{{\left( {1 - \alpha } \right)\left( {\alpha - \phi } \right)K_{f} + \phi K}}} \right]$$

and

12 $$\nu_{u} = \frac{{3\nu + B\left( {1 - 2\nu } \right)\left( {1 - \frac{K}{{K_{s} }}} \right)}}{{3 - B\left( {1 - 2\nu } \right)\left( {1 - \frac{K}{{K_{s} }}} \right)}}$$,

respectively.

For quasi-static phenomena additionally characterized by negligible body forces, the conservation of linear momentum becomes:

13 $${\text{div}} ({{\varvec{\upsigma}}}) = {\mathbf{0}}$$

The conservation of mass is written in the form:

14 $$\frac{\partial \zeta }{{\partial t}} + {\text{div}} ({\mathbf{q}}) = s$$

where $s$ is the eventual source density, that is the rate of injected fluid volume per unit volume of the porous solid.

By accounting for the Darcy’s law (5) and the expression for the fluid content (7), the conservation of mass equation (14) can be expressed in terms of the stress ${{\varvec{\upsigma}}}$ and the pressure $p$, leading to the following formula:

15 $$\kappa \,\nabla^{2} p + s = \frac{{3\left( {\nu_{u} - \nu } \right)}}{{2G\,B\left( {1 + \nu } \right)\left( {1 + \nu_{u} } \right)}}\frac{\partial }{\partial t}\left( {{\text{tr}} ({{\varvec{\upsigma}}}) + \frac{3}{B}p} \right)$$

By setting

$$c = \frac{{2GB^{2} \kappa \left( {1 - \nu } \right)\left( {1 + \nu_{u} } \right)^{2} }}{{9\left( {1 - \nu_{u} } \right)\left( {\nu_{u} - \nu } \right)}}~~~~~~g = \frac{{2GB\left( {1 + \nu } \right)\left( {1 + \nu_{u} } \right)}}{{3\left( {\nu_{u} - \nu } \right)}}$$

Eq. (15) can be also rewritten in the alternative equivalent form as:

16 $$c\nabla^{2} \left( {{\text{tr}} ({{\varvec{\upsigma}}}) + \frac{3}{B}p} \right) + g\,s = \frac{\partial }{\partial t}\left( {{\text{tr}} ({{\varvec{\upsigma}}}) + \frac{3}{B}p} \right)$$

### Proposal of isotropic remodeling influenced by fluid flow

In this work, a new remodeling formulation, aimed to extend the Stanford law proposed by Beauprè et al. (1990b) by including the dimensionless fluid content, is proposed. In more detail, by recalling that the bone tissue is a poroelastic medium, from Eq. (7), the dimensionless fluid content $\zeta$ can be expressed as

17 $$\zeta = \frac{\alpha }{2G}\frac{1 - 2\nu }{{1 + \nu }}\left( {{\text{tr}} ({{\varvec{\upsigma}}}) + \frac{3}{B}p} \right)$$

We thus assumed that the stress stimulus at tissue level $\psi_{t}$ is a function of the density $\rho$, the stress $\sigma_{i}$, and the fluid content $\zeta$ as well. In this way, the fluid content can be in particular interpreted as a triggering factor influencing both the supply of nutrients and the removal of wastes of bone tissue cells, in turn affecting the relation between stress stimulus at the tissue level $\psi_{t}$ and remodeling rate of density $\dot{r}$. This more realistic interpretation of the bone remodeling process can be translated mathematically by means of an ad hoc function $f_{\zeta }$, related to the dimensionless fluid content $\zeta$, as follows

18 $$\begin{array}{*{20}c} {{\text{if}}\quad \zeta \ge 0} & {f_{\zeta } = (1 + \zeta )^{\gamma } } \\ {{\text{if}}\quad \zeta < 0} & {f_{\zeta } = (1 + \left| \zeta \right|)^{ - \gamma } } \\ \end{array}$$

where the quantity $(1 + \zeta )$ works as an activator factor and $\gamma$ is the power to be set for properly describing the effectiveness of the action. The power value $\gamma$ depends in fact on the level of activities of the bone tissue cells, the concentration and quality of the fluid in terms of possible presence of growth factors, and needs to be determined by experimental tests. The function $f_{\zeta }$ is so a measure of the surplus of fluid content strictly related to the applied loads to which the bone tissue is subjected to. If $\zeta > 0$, the bone tissue has a surplus of fluid and, as a consequence, a greater supply of nutrients and a better capability to remove wastes of bone tissue cells. Conversely, if $\zeta < 0$, the dearth of the fluid reduces the amount of nutrients available for bone tissue cells and helps the stack of wastes. If $\zeta = 0$, the bone tissue does not involve a strain due to proroelastic effect and thus the bone tissue is soaked by basal fluid, and the effect of the activator factor vanishes.

The stress stimulus at tissue level $\psi_{t}$ described in eq. (2) is here reformulated to take into account the fluid content $\zeta$ through the function $f_{\zeta }$, that is

19 $$\psi_{{t_{{_{proposal} }} }} = f_{\zeta } \psi_{t}$$

It is worth to precise that the proposed modeling approach, analogously to that based on the standard Stanford’s law, is a macroscopic one. This implies that absorption and resorption activities occurring at the microscopic level and determined at that scale by the competition between osteoblasts and osteoclasts, are somehow “projected” at the continuum level where, in fact, mechanical stress and fluid content (supplying nutrients in the bone districts) all contribute with the cells’ dynamics to the macroscopic bone density upshot. This means that the macroscopic outcomes do not simply “copy” what happens at the microscale among bone cells, the remodeling being the final result of processes occurring across the scales (Fraldi and Carotenuto 2018).

The adopted remodeling parameters in (3), i.e., the resorption slope $c_{r}$, the formation slope $c_{f}$, and the stress stimulus reference value $\psi_{ref}$ are material constants, estimated from experimental studies present in literature (Beauprè et al., 1990b) and are listed in Table 1.

**Table 1:** Remodeling material parameters

Remodeling material parameters		
Reference stress stimulus	$\Psi$_ref_ [MPa/day]	50
Loads number of cycles	n [cycles/day]	6000
Load weighting factor	m	4
Formation slope	c_f_ [mm/day]	0.02
Resorption slope	C_r_ [mm/day]	0.02
Homeostasis window width	w [MPa/day]	25

**Table 2:** Poroelastic material parameters

Poroelastic material parameters		
Solid phase bulk modulus	K_s_ [MPa]	16,000
Fluid phase bulk modulus	K_f_ [MPa]	2300
Poisson’s coefficient	$\nu$	0.25
Permeability	K [mm^2^/MPa s]	656

**Fig. 2:**
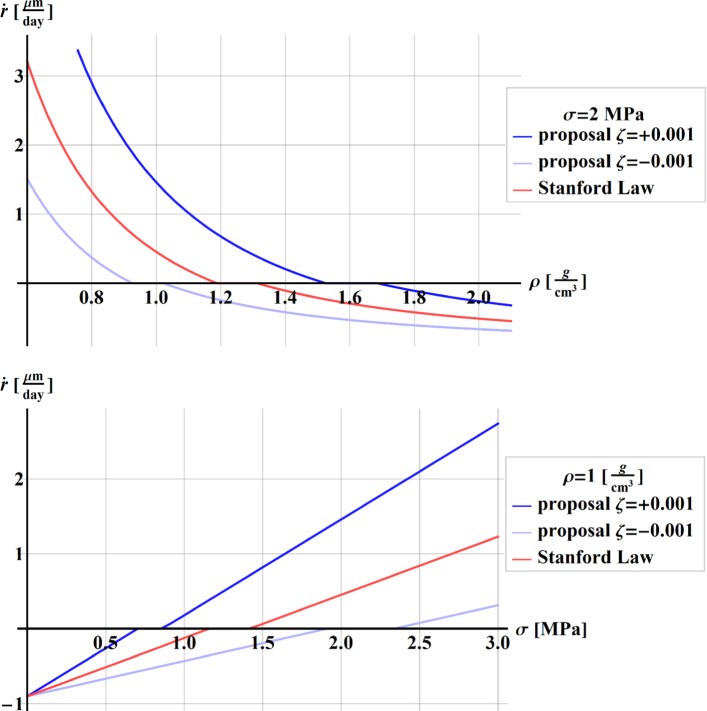
Comparison in terms of remodeling rate $\dot{r}$ between Stanford law (red) and our proposal, by setting the fluid content $\zeta = + 0.001$(darker blue) and $\zeta = - 0.001$ (lighter blue), by keeping fixed the stress to the value $\sigma = 2\,{\text{MPa}}$ (up), and by keeping fixed the density to the value $\rho = 1\;{{\text{g}} \mathord{\left/ {\vphantom {{\text{g}} {{\text{cm}}^{3} }}} \right. \kern-\nulldelimiterspace} {{\text{cm}}^{3} }}$ (down)

By using these values, the comparison in terms of remodeling rate $\dot{r}$ between the Stanford law (red line) and our proposal is shown in Fig. 2, by keeping fixed the level of stress to $\sigma = 2\;{\text{MPa}}$ (up) and the value of the density to $\rho = 1\;{{\text{g}} \mathord{\left/ {\vphantom {{\text{g}} {{\text{cm}}}}} \right. \kern-\nulldelimiterspace} {{\text{cm}}}}^{3}$ (down). The lighter and darker blue lines refer to the poroelastic formulation with a surplus ($\zeta = + 0.001$) and a lack ($\zeta = - 0.001$) of fluid content, respectively (Table 2).

It is worth noting that, in the case of bone formation and oversupply of fluid content, that is $\zeta = + 0.001$ corresponding to the darker blue lines, the stress at tissue level and, consequently, the rate of the bone remodeling result higher than that predicted by the Stanford law. On the other hand, in the case of bone absorption, the bone tissue will resorb less. These effects result in complementary ones (lighter blue line) if there is a lack of fluid content, that is $\zeta = - 0.001$. For example, as it can be noted in Fig. 2 (up), if $\rho = 0.8\;{{\text{g}} \mathord{\left/ {\vphantom {{\text{g}} {{\text{cm}}^{3} }}} \right. \kern-\nulldelimiterspace} {{\text{cm}}^{3} }}$, the corresponding value of the rate of bone formation obtained with Stanford law is 1.32 ${{\mu m} \mathord{\left/ {\vphantom {{\mu m} {day}}} \right. \kern-\nulldelimiterspace} {day}}$ (red line). With a surplus of fluid content (darker blue line), this value rises to 2.89 ${{\mu m} \mathord{\left/ {\vphantom {{\mu m} {day}}} \right. \kern-\nulldelimiterspace} {day}}$ (+ 120%), and goes down to 0.37 (− 70%) with a lack of the fluid content (lighter blue line).

In the case of homeostasis, the effect of the fluid content on the remodeling rate acts modifying the DZ window width, and both the upper and lower values of the homeostasis region. It is worth to highlight that, for densities corresponding to DZ for Stanford law, our formulation furnishes values of rate of bone remodeling corresponding to formation ($\zeta = + 0.001$) or resorption ($\zeta = - 0.001$).

Finally, the assumed remodeling rate relation was supposed to provide a zone of saturation. The saturation is present in both formation and absorption zones and is related to the yield stress limit $\sigma_{Y}$ of the material, set as a power function of the density $\rho$ of the material (Lotz et al., 1990), as

20 $$\sigma_{Y} = 25\rho^{1.8}$$

As a consequence, the daily stress stimulus $\psi$ measured at the continuum level in equation (1) was modified as

21 $$\begin{array}{*{20}c} {{\text{if}}\;\sigma_{i} < \sigma_{Y} ,\quad \psi = \left( {\sum\limits_{day} {n_{i} \overline{\sigma }_{i}^{m} } } \right)^{\frac{1}{m}} } \\ {{\text{if}}\;\sigma_{i} \ge \sigma_{Y} ,\quad \psi = \left( {\sum\limits_{day} {n_{i} \overline{\sigma }_{Y}^{m} } } \right)^{\frac{1}{m}} } \\ \end{array}$$

### Some paradigmatic examples

Two examples of direct interest in biomechanical applications have been chosen to verify the feasibility and the effectiveness of the proposed remodeling approach. To this purpose, both the classical Stanford Law and our proposal have been implemented by means of customized algorithms in the numerical Finite Element code ANSYS® Multiphysics (ANSYS Inc., Canonsburg, PA, USA) and the obtained results have been compared.

The first example is constituted by a beam with a rectangular section undergoing bending moment applied to the extremities, chosen to simulate, for instance, the behavior of a single trabecula or a cortical tract of bone tissue. The beam has a length of 20 mm and a rectangular section of 1 mm × 2 mm. Due to symmetry, only half of the structure has been analyzed. A bending moment equal to $0.26$ Nmm was applied to the ends of the beam and a vanishing flow has been also imposed on the external surfaces of the poroelastic structure.

The second example is constituted by a bi-phase hollow cylinder subject to prescribed radial displacements at the innermost boundary, in principle replicating an ideally cylindrical section of the diaphysis of the femur. The cylinder has a height equal to 200 mm, and the internal and the external radii have been set to 8.5 and 18 mm, respectively (Esposito et al., 2018, 2020). The thickness of the internal and external phases, i.e., the trabecular and cortical bone, have been assumed equal to 9.5 and 3 mm, respectively. Internal prescribed radial displacement equal to 0.4 mm has been applied to the internal faces of the cylinder in order to simulate the action of the prosthesis inside the femur canal. Also in this case, a vanishing flow has been imposed on the external surfaces of the structure.

**Fig. 3:**
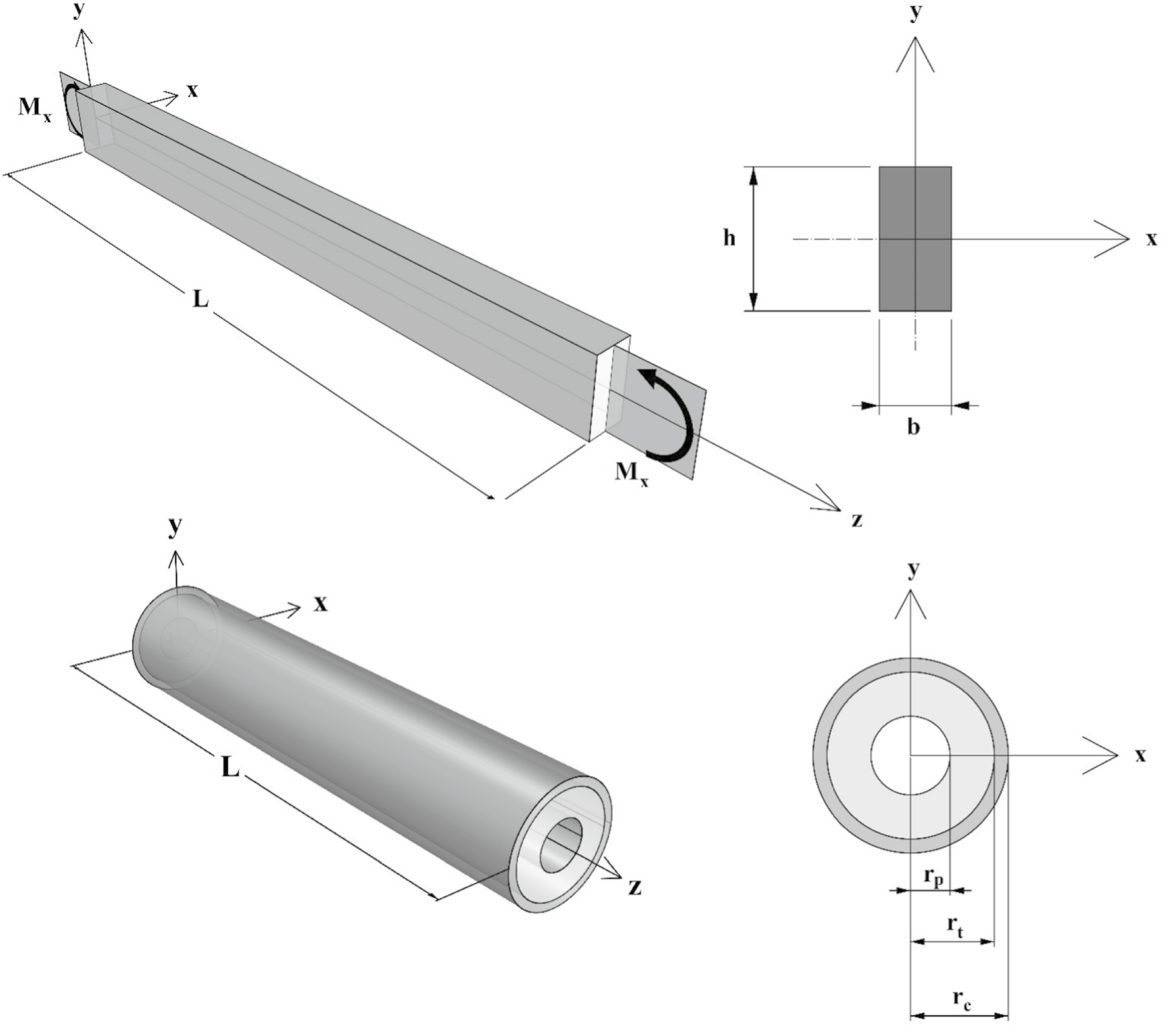
Geometry and boundary conditions of the considered examples. Beam with rectangular cross section subject to bending moment at the extremities (up). Bi-phase hollow cylinder subject to internal radial prescribed displacements (down)

A scheme of the geometries of the proposed structures is depicted in Fig. 3, e.g., the beam subject to bending moment (up) and the bi-phase hollow cylinder subject to internal radial prescribed displacements (down).

When implementing the poroelastic numerical simulations, porous media containing fluid are modeled into the FE code as a multiphase material, by applying an extended version of Biot’s consolidation theory. The flow is considered to be a single-phase fluid and the porous media is assumed to be fully saturated.

In order to implement our coupled fluid–structure remodeling formulation, the finite element identified in ANSYS by the code CPT215 has been chosen. The adopted element is a three-dimensional coupled pore-pressure mechanical solid element with eight nodes and linear shape functions. Four degrees of freedom are considered at each corner node, three translations in the nodal x, y, and z directions, and one pore-pressure degree of freedom. The typical form of the governing equations for Biot’s consolidation problems implemented in FEM code is:

22 $$\alpha^{(i)} \dot{\varepsilon }_{V} + \frac{1}{{M^{(i)} }}\dot{p} + \kappa^{(i)} \nabla^{2} p = s$$

where $s$ is an external source, supposed to be null in the present case, $\varepsilon_{V}$ is the volumetric strain and $M^{(i)}$ refers to the Biot’s moduli of the $i$-th material, one for each element of the meshed structure, being $i \in [1,..,N_{elems} ]$, and $N_{elems}$ the number of the elements.

With the aim of obtaining the FEM results, the equation (15) needs to be re-arranged according to the expression given in the equation (22).This aim is reached by setting the moduli $M^{(i)}$ as:

23 $$M^{(i)} = \frac{{E^{(i)} B^{{(i)^{2} }} \left( {1 + \nu_{u}^{(i)} } \right)}}{{9\left( {\nu_{u}^{(i)} - \nu^{(i)} } \right)}} = \frac{{E\left( {\nu_{u}^{(i)} - \nu^{(i)} } \right)}}{{\alpha^{{(m)^{2} }} \left( {1 - 2\nu^{(i)} } \right)^{2} \left( {1 + \nu_{u}^{(i)} } \right)}}$$

and the permeability modulus $\kappa^{(i)}$ as:

24 $$\kappa^{(i)} = \frac{{k^{(i)} }}{{\mu^{(i)} }}$$

The adopted poroelastic material parameters are listed in Table 1 (Cowin 1999), being $K_{s}$ and $K_{f}$ the bulk moduli of the solid and fluid phases, respectively, and $\kappa$ and $\nu$ the permeability moduli (24) and the Poisson’s coefficient, supposed to be prescribed for all elements.

Although the procedure would allow to deal with anisotropic materials, to highlight the effects of the fluid flow on the outcomes, the poroelastic media has been here supposed to be simply isotropic. The bulk modulus for each $i$-th element of the meshed structure has been set as a function of the porosity by means of a power value $q = 3$, that is

25 $$K^{(i)} = K_{s} (1 - \phi )^{q}$$

being the procedure open to host a different constitutive law.

## Results and discussion

The first considered example structure, i.e., the beam subject to bending moment, has been meshed by 2593 elements and 3325 nodes. The material of the beam has been set with a starting uniform distribution of density equal to $0.6\;{g \mathord{\left/ {\vphantom {g {cm^{3} }}} \right. \kern-\nulldelimiterspace} {cm^{3} }}$, with the aim of simulating bone tissue with an average density. The adopted material parameters, i.e., the porosity, the bulk modulus, and the Young modulus, are listed in Table 3.

**Table 3:** Starting material parameters for trabecular and cortical bone tissue

		Starting material parameters	
		Trabecular bone	Cortical bone
Density	$\rho$[g/cm^3^]	0.6	2.1
Bulk modulus	K [MPa]	290	14,000
Young modulus	E [MPa]	435	21,230

In the case of the second proposed example, i.e., the cylinder subject to internal radial prescribed displacements, the structure has been meshed by 2304 elements and 2704 nodes. In order to reduce the computational time, the mesh has been refined only in the central part of the cylinder and the internal trabecular and external cortical phases have been set with a starting uniform distributions of densities equal to $0.6$ and $2.1{g \mathord{\left/ {\vphantom {g {cm^{3} }}} \right. \kern-\nulldelimiterspace} {cm^{3} }}$, respectively, with the aim of replicating the features observed for the section of femoral diaphysis. The corresponding porosity, the bulk modulus, and the Young modulus of the trabecular and cortical tissue are still listed in Table 3.

The numerical simulations have been performed with the aim of simulating the remodeling process during a period of 360 days. At the end of each iteration, once calculated the current value of the density for each element, the corresponding material properties have been consequently updated by using the Eq. (25).

Sensitivity analyses have been performed by refining mesh in the range of half and double of the adopted element size. As the mesh size approached decreasing values, the obtained results showed increased accuracy and strong convergence for the outcomes from refined models. Furthermore, in order to avoid possible inaccuracy or concealed errors due to cumulative effects related to the continuous updating, at each iteration, of the remodeled bone poroelastic properties, whose spatial gradients occurring at a given step of the remodeling could imply a mesh refining unnecessary at early stages of the analyses, some code lines were incorporated in the proposed remodeling algorithm to check that, at each step, the reorganization and the reassignment of the material properties were compatible with the mesh size established at the starting point of the procedure, forcing to update and refine locally the no longer appropriate mesh size if density and/or associated stress gradients appeared too high at a certain step wherever in the model.

**Fig. 4:**
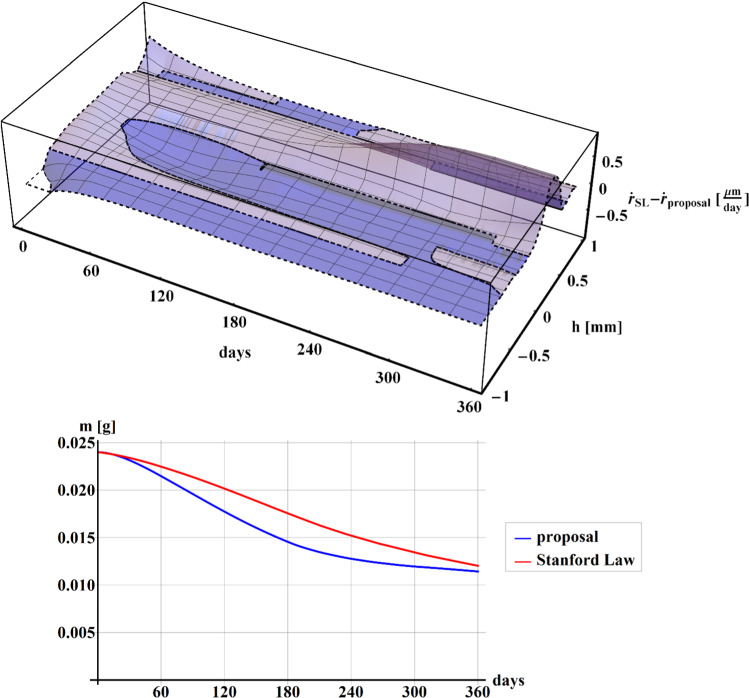
Plot of the difference between the rate of density $\dot{r}$ along with the section $h$, calculated when implementing the Stanford law and our proposal (up) during the 1-year time of simulation. Plot of the comparisons of the mass $m$ of the structure (down) for Stanford law (red line) and our proposal (blue line) during the 1-year time of simulation

In Fig. 4, the difference between the rates of density $\dot{r}$ (up) along with the section $h$, and the comparisons of the results for the mass $m$ of the structure (down) in case of Stanford law (red line) and our proposal (blue line), have been shown during the 1-year time of simulation.

It can be noted that the mass tendencies (see Fig. 4 down) result quite similar to that obtained by Stanford law (red line), with the highest difference of values at about 180 days. Despite the mass trend is comparable, it is worth to highlight that the surface representing the difference between the rate of density $\dot{r}$ calculated when implementing the Stanford law and our proposal (see Fig. 4 up) results as asymmetrical along with the section of the beam with higher values where the beam is in tension (positive $y$). The remodeling phenomenon tends to stabilize after 60–120 days for both formulations, in accordance with the data present in the literature.

**Fig. 5:**
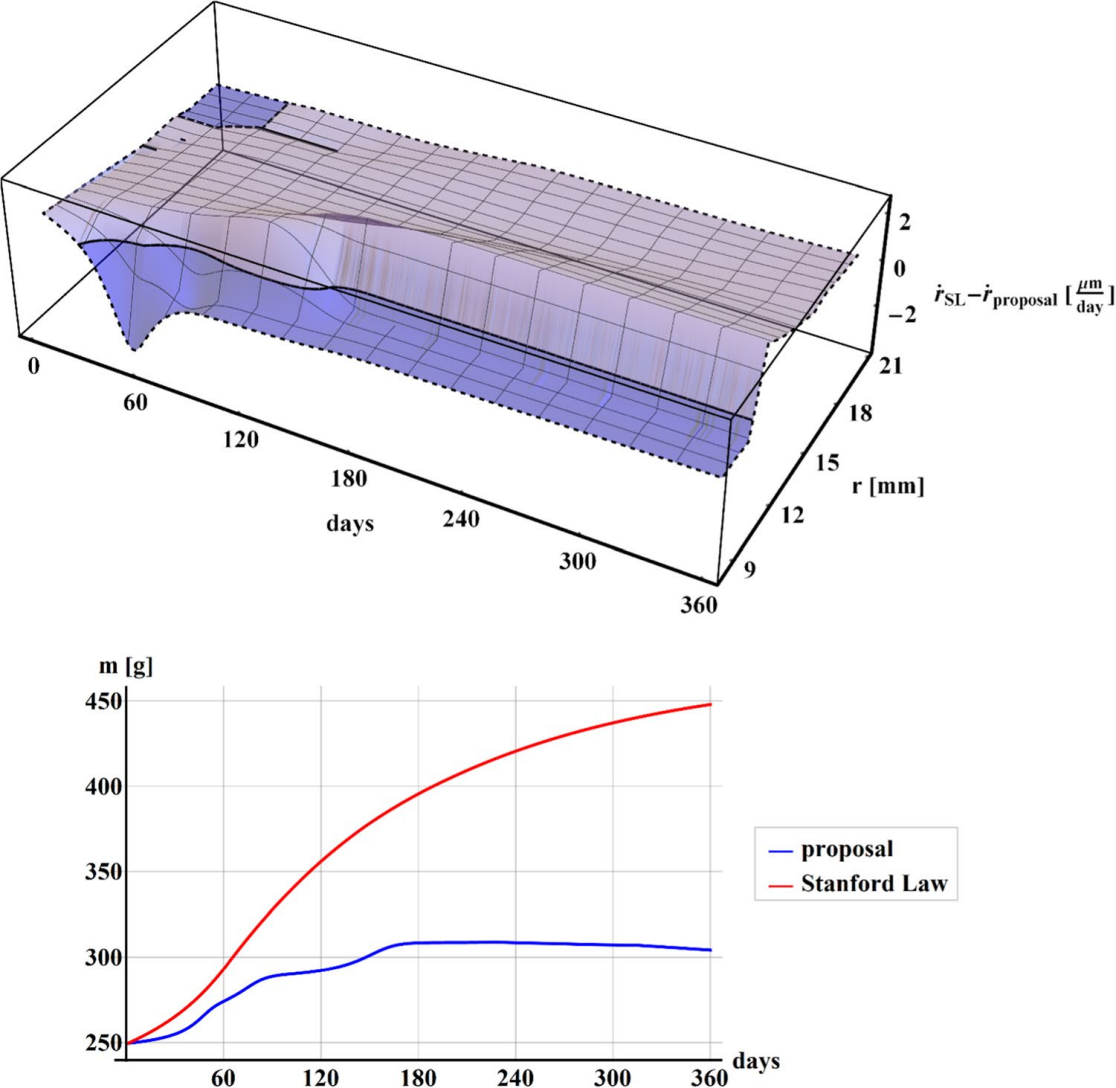
Plot of the difference between the rate of density $\dot{r}$ along the radius $r$, calculated when implementing the Stanford law and our proposal (up). Plot of the comparisons of the mass $m$ of the structure between Stanford law (red line) and our proposal (blue line), in the case of an hollow cylinder subject to internal pressure for 360 days

In Fig. 5, in the case of bi-phase hollow cylinder subject to internal radial prescribed displacements, the difference between the rate of density $\dot{r}$ (up) along with the radius $r$ and the comparisons of the mass $m$ of the structure (down) between Stanford law (red line) and our proposal (blue line), have been shown over the time period of 360 days.

In the case of the second proposed example, the mass obtained by our proposal (blue line) results much less than that obtained by Stanford law (red line) with a perceptual difference of about 33% at 360 days, as shown in the graph in Fig. 5 (down). Moreover, the difference between the rate of density $\dot{r}$ (up) tends to stabilize after 120 days showing values about $2\;{{\mu m} \mathord{\left/ {\vphantom {{\mu m} {{\text{day}}}}} \right. \kern-\nulldelimiterspace} {{\text{day}}}}$ for both trabecular and cortical phase, according to the data present in literature.

### The effect of the bending moment

The applied bending moment produced zones where the fluid squeezes (the zone subject to compression) and others where the fluid accumulates (where the beam is in tension). As it can be argued from Fig. 3, the bending moment was applied in such a way to have the upper fibers of the beam, corresponding to the positive $y$-axis, compressed. As a consequence of Darcy’s law, the fluid moved from the upper part (positive *y*-axis) to the lower part of the section (negative *y*-axis) of the beam.

As an indicator of the differences obtained by implementing the remodeling formulations, the perceptual difference of the density $\Delta \rho \%$ along with the height of the section has been taken into account as a reference, following the formula

26 $$\Delta \rho \% = \frac{{\rho_{{\text{Stanford Law}}} - \rho_{{{\text{proposal}}}} }}{{\rho_{{\text{Stanford Law}}} }}\%$$

**Fig. 6:**
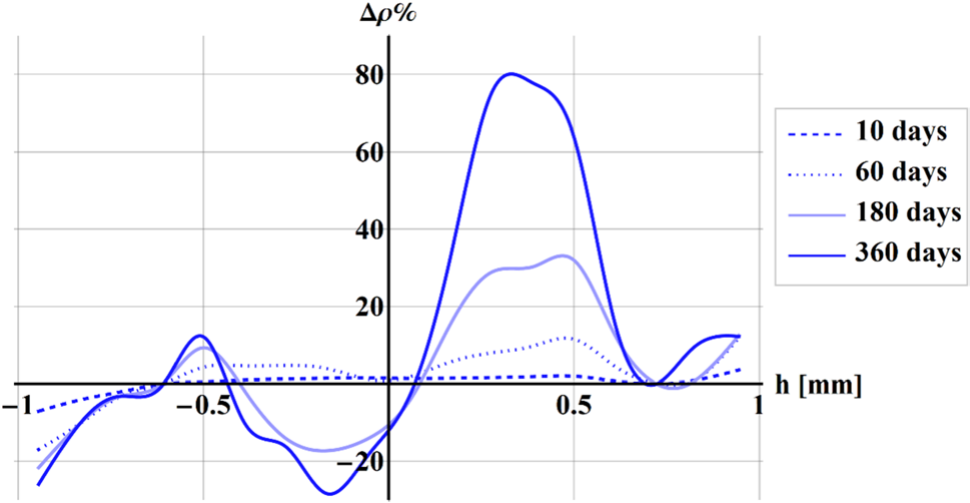
Plot of the perceptual difference of the density between Stanford law and our proposal along with the height of the beam at 10 days (dotted line), 60 days (dashed line), 180 days (lighter blue line) and 360 days (darker blue line)

Figure 6 shows the perceptual difference of the density $\Delta \rho \%$ along with the height of the beam at 10 days (dotted line), 60 days (dashed line), 180 days (lighter blue line), and 360 days (darker blue line).

Over the time period of 360 days, the negative and positive maximum values of $\Delta \rho \%$ are placed in the zones where the beam was extended and compressed, respectively, as a consequence of the fluid moving from the upper to the lower part of the section.

**Fig. 7:**
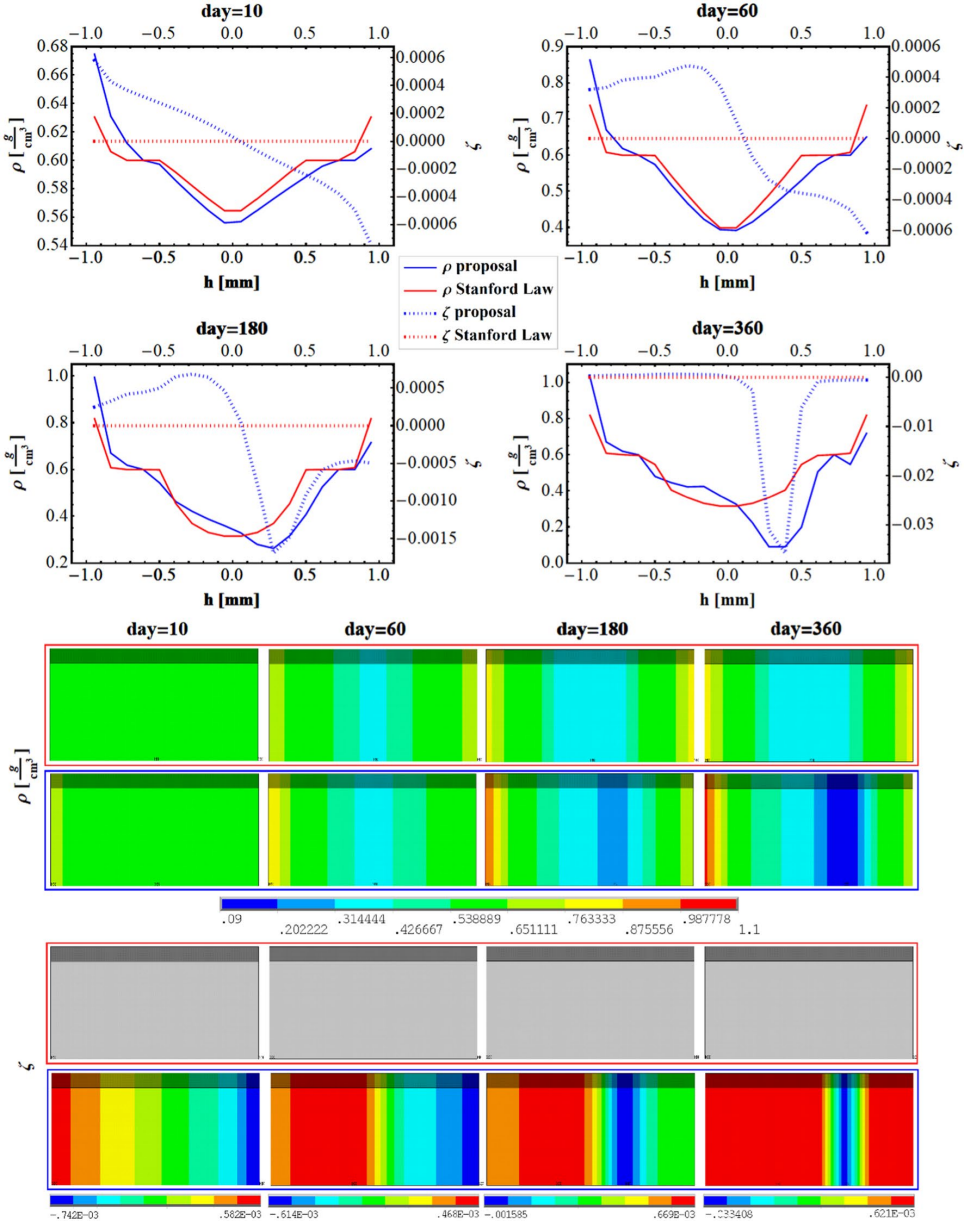
Plot of the density (continuous) and fluid content (dotted) when implemented Stanford law (red) and our proposal (blue), along with the height of the beam at 10 days 60 days, 180 and 360 days. Contour plots of the density and fluid content when implemented Stanford law (upper row) and our proposal (lower row), along with the height of the beam at 10 days 60 days, 180 and 360 days

In the upper part of Fig. 7, the density (continuous line) and the fluid content (dotted line) along with the height of the beam are plotted when implemented Stanford law (red) and our proposal (blue), at 10 days, 60 days, 180 days, and 360 days. The corresponding contour plots are shown in the lower part of Fig. 7.

As an effect to the applied bending moment, in the case of both remodeling models, the material grows on the outer surfaces and absorbs in the central part of the beam. The density distribution produced by using the Stanford law (continuous red line) remains symmetrical during the time with highest and equal values at the outermost fibers. On the contrary, it is worth to highlight that the proposed formulation, due to the direct effect of the fluid content—moved by pressure gradients and driving nutrients—leads to have asymmetry of the results in terms of spatial distribution of density during remodeling (continuous blue line). Also, the presence of the fluid varies the values of the stress stimulus in both absorption and formation phases. In fact, the comparison with the Stanford law model reveals that, in the zones where the beam undergoes tensile regime, the dimensionless fluid content results as positive and, in turn, the values of the rate of density are higher than that produced by Stanford law, with the final effect of denser material in the lower part of the beam and rare in the upper zones. Also, the stress stimulus at tissue level and the rate of remodeling resulted as asymmetrical when implementing our poroelastic model. In particular, the presence of the fluid content enlarged the zone where the density reached lower values, as well as the zone where the stress and, as a consequence, the stress stimulus were low. This can be explained by the Eq. (1), where the stress stimulus depends on the local effective stress $\overline{\sigma }_{i} = \sqrt {2EU_{i} }$, being this measure of the stress always positive. As a consequence, the Stanford law is somehow transparent to the sign of the stress, being unfit to identify the zone where the beam is tensed or compressed and hence distributing the same amount of material independently from the sign of the stress. Noteworthy, the introduction of the poroelastic approach in the remodeling formulation brings out the sign of the stress, the fluid content playing the role of a mechanosensing mediator and so orienting asymmetrically the deposition/resorption process.

**Fig. 8:**
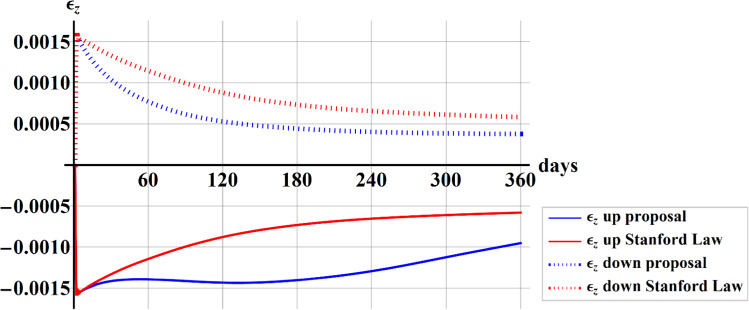
Plot of the comparison of the $z$-components of the strain calculated in the upper and lower height section of the beam between Stanford law (red) and our proposal (blue) has been shown during 360 days

In Fig. 8, the comparison of the $z$-component of the strain calculated in the upper (dotted) and lower (continuous) height section of the beam taking into account Stanford law (red) and our proposal (blue), has been shown during 360 days.

The $z$-components of the strain resulted also asymmetrical when implementing our proposal (blue line) and the presence of the fluid significantly modifies the trend during the time, with greater absolute values in the lower part of the section (continuous line) subject to tension.

### The effect of internal radial prescribed displacements

**Fig. 9:**
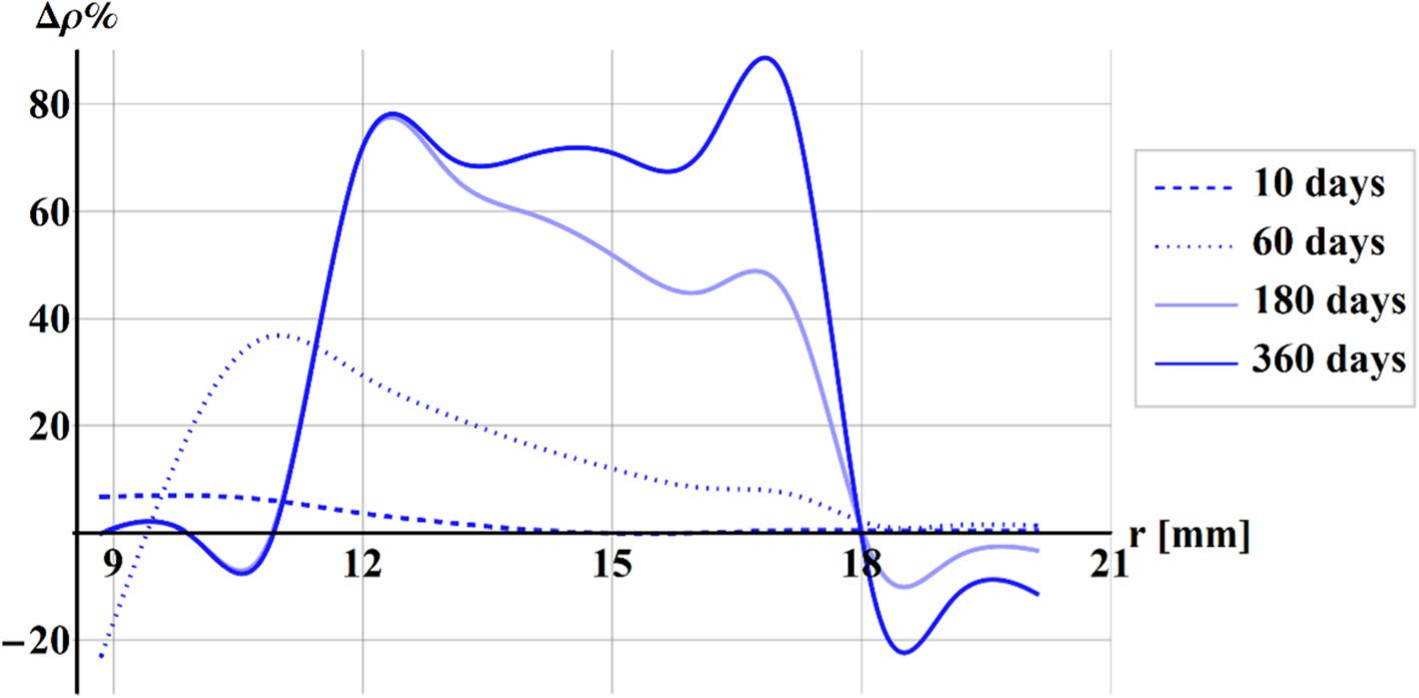
Plot of the perceptual difference of the density between Stanford law and our proposal along with the radius of the cylinder at 10 days (dotted line), 60 days (dashed line), 180 days (lighter blue line), and 360 days (darker blue line)

Figure 9 shows the perceptual difference of the density $\Delta \rho \%$ between Stanford law and our proposal along with the radius of the cylinder at 10 days (dotted line), 60 days (dashed line), 180 days (lighter blue line), and 360 days (darker blue line).

The maximum $\Delta \rho \%$ resulted to be about + 6% at 10 days at the internal radius. During the time, the position along with the radius of the maximum perceptual difference moves toward the external cylinder, with values about 40% at 60 days, almost 80% at 180 days, and more than 80% at 360 days.

**Fig. 10:**
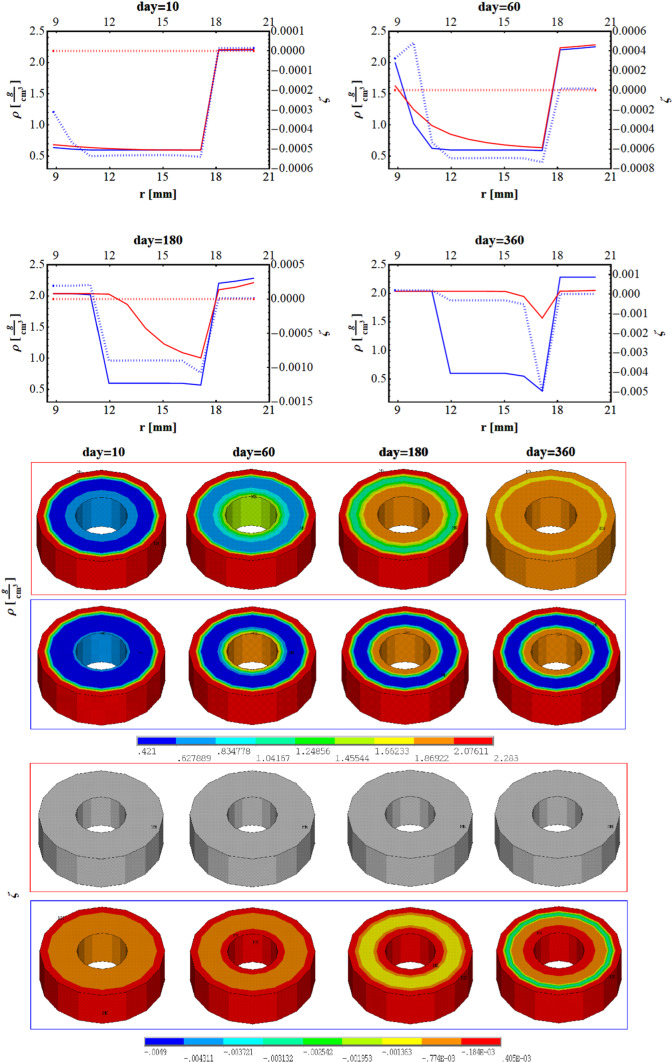
Plot of the density (continuous) and fluid content (dotted) when implemented Stanford law (red) and our proposal (blue), along with the height of the beam at 10 days 60 days, 180, and 360 days. Contour plots of the density and fluid content when implemented Stanford law (upper row) and our proposal (lower row), along with the height of the beam at 10 days 60 days, 180 and 360 days

In the upper part of Fig. 10, the density (continuous line) and the fluid content (dotted line) along with the radius of the cylinder are plotted when implemented Stanford law (red) and our proposal (blue), at 10 days, 60 days, 180 days, and 360 days. The corresponding contour plots are shown in the lower part of Fig. 10.

Independently from the specific numerical values implemented in the analyses and obtained as results, it is worth to notice that the outcomes show that both formulations gave a thickening of the material up to the cortical value at the internal radius, however, in a faster way for the poroelastic model. Additionally, when implementing our formulation (continuous blue line), the density became quite symmetrical at 180 days with cortical density values at the internal and external radius. Conversely, when implementing the Stanford law, the density reached the cortical value for most of the section.

**Fig. 11:**
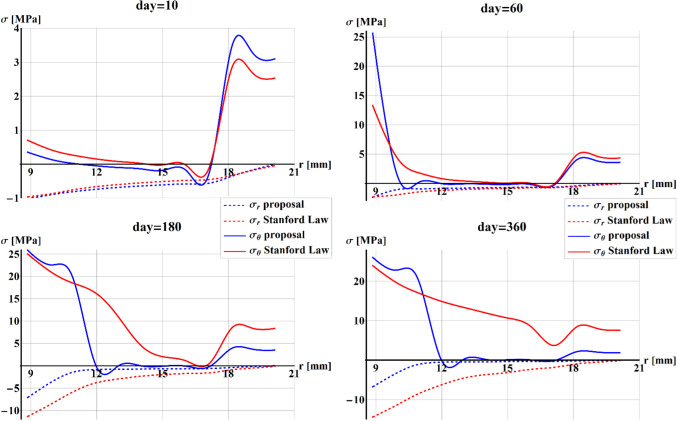
Plot of the comparison between Stanford law (red) and our proposal (blue) of the cylindrical in-plane components of the stress $\sigma_{r}$ (dotted line) and $\sigma_{\vartheta }$ (full line), along with the radius of the cylinder at 10 days, 60 days, 180 days, and 360 days

Figure 11 shows the comparison between Stanford law (red) and our proposal (blue) of the cylindrical in-plane components of the stress, $\sigma_{r}$ (dotted line) and $\sigma_{\vartheta }$ (full line), plotted along with the radius of the cylinder at 10 days, 60 days, 180 days, and 360 days.

The stress along with $r$ resulted as always negative due to the compression prescribed by the internal radial displacements, and with a null value at the external radius due to boundary conditions. After 10 days, $\sigma_{r}$ was similar for both formulations. During the time, when implementing our proposal (blue line), the presence of the fluid produced lower negative values of the stress along with $r$.

The circumferential stress resulted as similar for both formulations at 10 days, and at 60 days showed a higher value at the internal radius when implementing our proposed model. At 180 and 360 days, the circumferential stress values became again similar at the inner cylinder, reaching different distributions along with $r$ and values close to zero toward the outer radius when implementing our proposal. Therefore, also for this case, some qualitative discrepancies were registered by adopting the two models and the effect of the nutrients moved by the fluid flow influenced significantly the entire remodeling process, both in terms of in time and spatial distribution of mass density and stresses.

#### A real study case for testing the proposed model

**Fig. 12:**
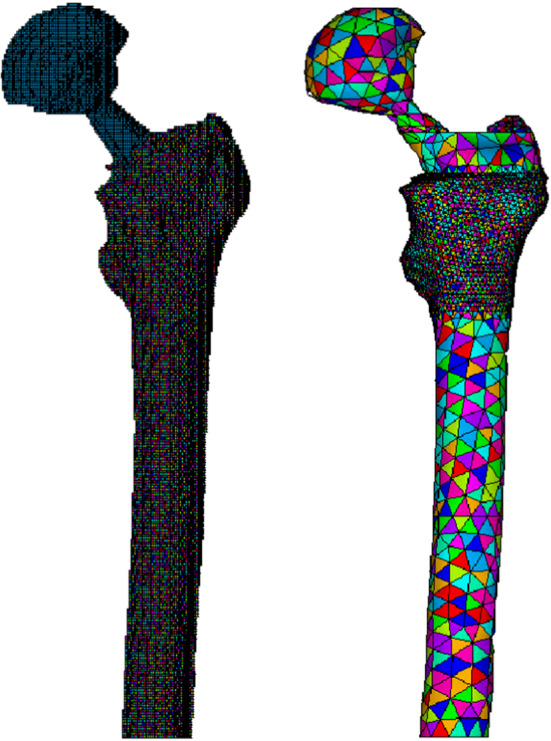
The voxel-based (left) and geometry-based (right) models

A clinical trial at the Healthcare Center of the Icelandic National Hospital (Landspitali, Department of Science, Education and Innovation (DSEI) and Department of Orthopaedic Sciences) was launched, and a cohort of 36 patients undergoing primary Total Hip Arthroplasty (THA) was systematically monitored in the framework of an international research project. The clinical trial provided to scan patients by means of a 64-slice spiral Computed Tomography (CT) Philips Brilliance scanner three times in one year: before THA, immediately after surgery (24H), and finally at 52 weeks post-surgery. The CT scanning region started from the iliac crest and ended at the middle of the femur; the slice thickness was 1 mm, the slice increment was 0.5 mm, and the tube voltage was set to 120 kV, allowing for precise 3D reconstructions of the regions of interest (Ricciardi et al., 2020). As known, Computed Tomography is a methodology used to acquire volumetric densities of tissues. In principle, there are two basic concepts of generating Finite Element (FE) models from CT data: geometry-based and voxel-based. The voxel-based meshing technique is achieved by matching each CT voxel to a single finite element, and the main advantage of this strategy is that it is a simple and automated technique. However, curved and smooth surfaces cannot be properly represented by brick elements, the jagged-edged surface for instance causing peak stresses and strains, which thus constitutes a disadvantage when accurate mechanical data are needed at those surfaces as well. Moreover, unstable elements (i.e., elements insufficiently anchored to the whole model and thus potentially involved in partial rigid body motion) can be generated during the 3D reconstruction, which is a crucial problem in obtaining consistent FE models, hindering mechanical analyses (Esposito et al., 2016). A patient from the above-mentioned cohort has been considered, and the related CT data at 24 h have been used to build-up the corresponding in silico model by means of the voxel-based approach. The obtained mesh consisted of about 500 k elements and associated 500 k nodes. Due to the high computational times related to poroelastic-based remodeling analyses, the geometry-based meshing strategy was chosen, the extraction of the outer contours of bone and prosthesis from CT scans being obtained by applying a related Hounsfield Unit (HU)-based filter. Then, femurs were 3D modeled and meshed using standard 10-node tetrahedral elements (three degrees of freedom associated to each node, with quadratic shape functions) in Ansys environment. The element size was set to 2 mm in the region of major interest, i.e,. the trochanter zone, while the element mean size of 8 mm in the remaining part of the model was adopted. The obtained mesh so resulted of 70 k elements and associated nodes. In order to derive the mechanical properties of the bone from the CT scan data, CT numbers or HU values were first converted into bone densities by means of a phantom calibration (Quasar Multi-Purpose Body Phantom) with known material densities scanned with the patients. Then, bone material properties, both elastic and porous parameters, were estimated from these data. The model was loaded by forces on the prosthesis cup by considering the actions related to the living daily activities (Bergmann et al., 2001) and constrained in the distal part of the model. Both the classical Stanford’s law and our proposal were implemented in the FE-based algorithm, performing the numerical simulations for a period of 360 days, finally comparing the results of the two models. The density data extracted from the real bone at 1 year have been used as a benchmark. Figure 12 shows the numerical models obtained with the voxel-based (left) and the geometry-based (right) strategies.

**Fig. 13:**
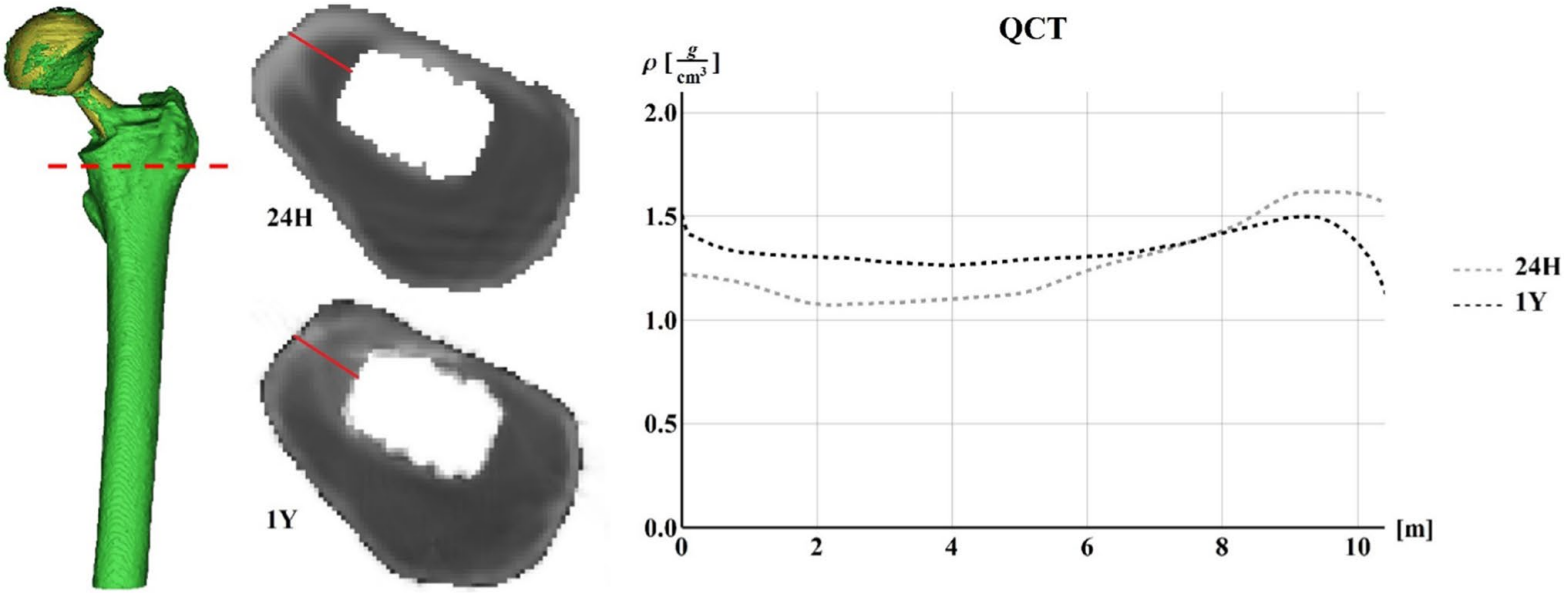
The whole femur (left), the maps of the actual density over the considered transverse sections in the trochanter region at 24H (top-middle) and 1Y (bottom-middle), and the corresponding plot of the densities obtained by CT data

Figure 13 shows the whole three-dimensional femur obtained by filtering CT data (left), the maps of the density of the considered transverse section at 24H (top-middle) and 1Y (bottom-middle), and the corresponding plot of the densities obtained by CT data along a line from the innermost to outermost points across a femur region actually interested by significant changes in bone density, with clear spatially inhomogeneous transition from cortical to trabecular tissues. Note that the dashed red line identifies the transverse section in the trochanter region, while the results have been calculated along the path described by the full red lines in the sections at the center. It is then worth highlighting the differences between the curves related to the density at 24H and 1Y. In particular, the density at 1Y grows up near the prosthesis with an increment of about 30% and an approximately 10% decrement in the bone’s outer part.

**Fig. 14:**
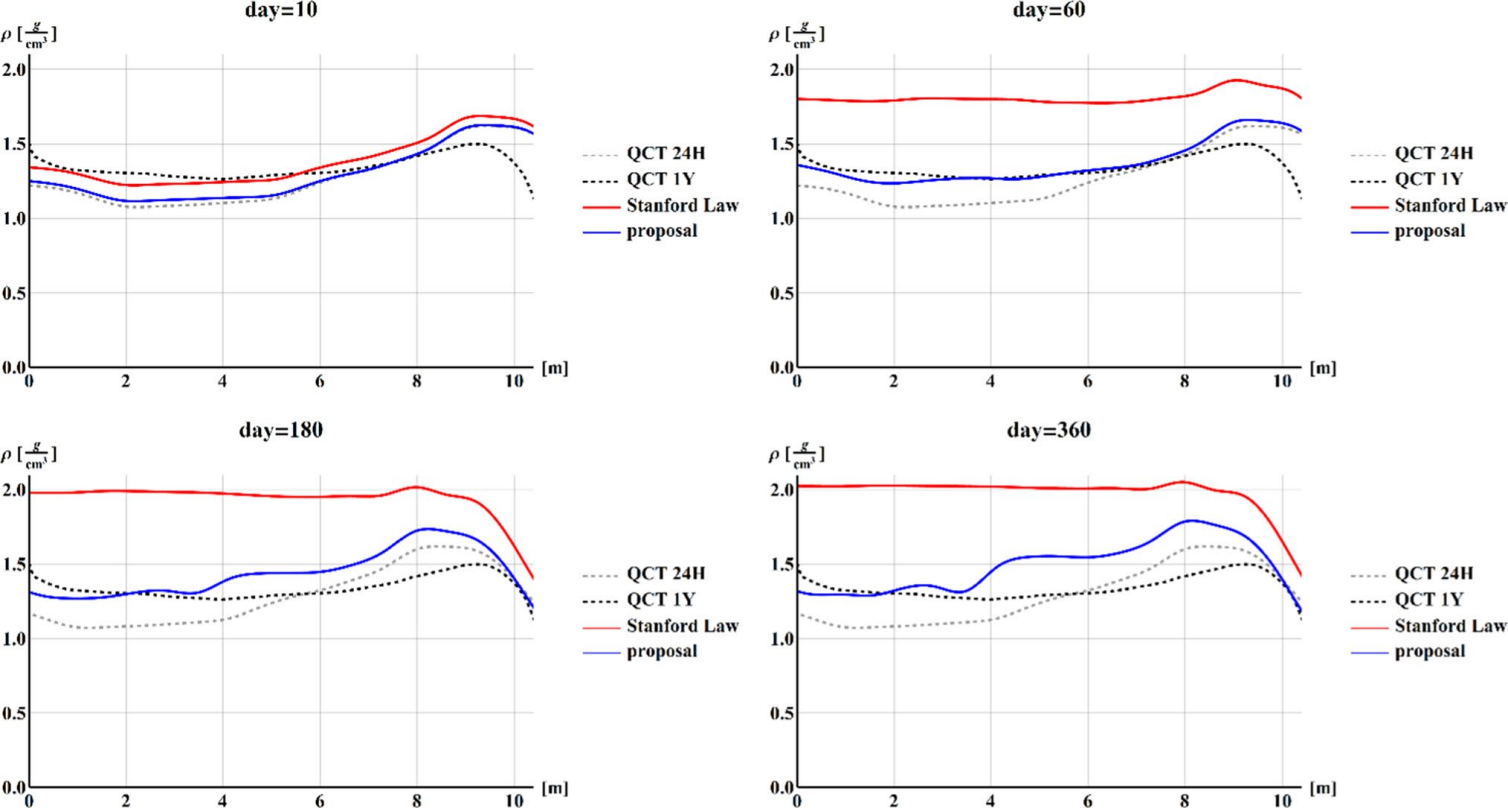
Plot reporting the comparison between predictions from Stanford law (red) and from our proposal (blue) of the bone densities along the transverse section of the femur in the trochanter region at 10 days, 60 days, 180 days and 360 days

The results are synthetically illustrated in terms of the bone densities. In particular, Fig. 14 shows the comparison between the predictions from Stanford law (red) and from our proposal (blue) as densities along the transverse section of the femur in the trochanter region at 10 days, 60 days, 180 days and 360 days.

The results show that both the model approaches produced a denser bone at the interface with the prosthesis. However, when implementing the Stanford law, the simulated remodeling process predicts values of density close to those of the cortical bone in the whole section, starting from the outcomes at 60 days and up to one year, in contrast to the actual density distribution one year after the implant. On the contrary, the proposed poroelastic-based remodeling strategy—which accounts for the role of pore pressure and fluid-driven nutrient walkway, in turn influencing the density rate – seems instead affect growth and remodeling, the densities curves replicating more faithfully the actual bone density profile measured at one year along the selected line of the femur slice of the benchmark.

It must be highlighted that, despite the very encouraging obtained results, the a priori setting of the crucial parameter *p* of the proposed model would deserve a deeper discussion, for example by designing an experiment to be performed in vitro, a topic that is, however, beyond the scope of the present work.

## Conclusions

The work presented an improved version of the classical so-called Stanford’s law, widely employed to predict the time-dependent evolution of the bone density in response to load-induced mechanical stimuli, mediated by the stress field ingenerated within the tissue day-by-day. By neglecting elastic anisotropy and with the aim of capturing some experimentally observed asymmetries in bone mass redistribution, even when in the presence of symmetrical stress states, we proposed to enrich the standard Stanford’s law by coupling the direct effect of the stress stimulus on bone growth with the spatially inhomogeneous nutrient supply kindled by pressure gradients inside the bone, modeled as a poroelastic medium. By following this way, stress and fluid flow synergistically allowed to reproduce medium/long-term asymmetries and more realistic outcomes in terms of mass rate and bone tissue remodeling. To highlight quantitative as well as qualitative differences in the remodeling outcomes when adopting the classical and the proposed models, two study cases, i.e., a ring under axis-symmetrical conditions and a plate under pure bending, were built up and numerically solved as the simplest paradigms of benchmark remodeling problems, which gave symmetrical results when adopting standard approaches based on Stanford’s law, giving instead non-symmetrical and biophysically coherent results in terms of bone density spatial distribution, if the classical Stanford’s law was enriched by taking into account the role of the fluid transporting nutrients throughout the poroelastic bone medium. These two examples, which somehow evoked the cross section of a femur pushed from the inner by the pressure exerted by a femur prosthesis stem and a tract of bone under classical bending regime as experienced in vivo during oscillatory loading, were therefore crucial to highlight, in the easiest way, the important capability of the proposed strategy of reproducing symmetry breaking of bone density distribution resulting from the cooperation of stress and fluids, not ever considered in previous studies.

Although limitations still characterize some hypotheses at the basis of the present approach, the proposed model overcomes the intrinsic—and unrealistic—independence of the bone remodeling from the stress sign and from the indirect effect of stress gradients driving nutrients through the flow of the fluid content in the tissue, allowing to predict important spatial asymmetries in bone mass density, so paving the way to more reliable mechanobiological strategies and engineering tools for the faithful prediction of bone remodeling, with implications in diagnosis of risk fracture, optimal design of bone prostheses, and precise medicine.
